# Clinical characteristics, causes and predictors of outcomes in patients with in-hospital cardiac arrest: results from the SURVIVE-ARREST study

**DOI:** 10.1007/s00392-022-02084-1

**Published:** 2022-08-17

**Authors:** Laura Erika Maria Hannen, Betül Toprak, Jessica Weimann, Bahara Mahmoodi, Nina Fluschnik, Benedikt Schrage, Kevin Roedl, Gerold Söffker, Stefan Kluge, Malte Issleib, Stefan Blankenberg, Paulus Kirchhof, Peter Clemmensen, Christoph Sinning, Elvin Zengin-Sahm, Peter Moritz Becher

**Affiliations:** 1 Department of Cardiology, University Heart and Vascular Centre Hamburg, University Medical Centre Hamburg-Eppendorf, Martinistrasse 52, 20246 Hamburg, Germany; 2 German Centre for Cardiovascular Research (DZHK), Partner site Hamburg/Kiel, Lübeck, Germany; 3 Department of Intensive Care Medicine, University Medical Centre Hamburg-Eppendorf, Hamburg, Germany; 4 Centre of Anaesthesiology and Intensive Care Medicine, University Medical Centre Hamburg, Hamburg, Germany; 5 Institute of Cardiovascular Sciences, University of Birmingham, Birmingham, UK; 6 Department of Regional Health Research, Faculty of Health Sciences, University of Southern Denmark and Nykoebing Falster Hospital, Odense, Denmark; 7 Adult Congenital Heart Disease Section, University Heart and Vascular Centre Hamburg, Hamburg, Germany

**Keywords:** In-hospital cardiac arrest, Hospitalization, Outcomes, Survival, Mortality

## Abstract

**Introduction:**

In-hospital cardiac arrest (IHCA) is acutely life-threatening and remains associated with high mortality and morbidity. Identifying predictors of mortality after IHCA would help to guide acute therapy.

**Methods:**

We determined patient characteristics and independent predictors of 30-day in-hospital mortality, neurological outcome, and discharge/referral pathways in patients experiencing IHCA in a large tertiary care hospital between January 2014 and April 2017. Multivariable Cox regression model was fitted to assess predictors of outcomes.

**Results:**

A total of 368 patients with IHCA were analysed (median age 73 years (interquartile range 65–78), 123 (33.4%) women). Most patients (45.9%) had an initial non-shockable rhythm and shockable rhythms were found in 20.9%; 23.6% of patients suffered from a recurrent episode of cardiac arrest. 172/368 patients died within 30 days (46.7%). Of 196/368 patients discharged alive after IHCA, the majority (72.9%, *n* = 143) had a good functional neurological outcome (modified Rankin Scale ≤ 3 points). In the multivariable analysis, return of spontaneous circulation without mechanical circulatory support (hazard ratio (HR) 0.36, 95% confidence interval (CI) 0.21–0.64), invasive coronary angiography and/or percutaneous intervention (HR 0.56, 95% CI 0.34–0.92), and antibiotic therapy (HR 0.87, 95% CI 0.83–0.92) were associated with a lower risk of 30-day in hospital mortality.

**Conclusion:**

In the present study, IHCA was survived in ~ 50% in a tertiary care hospital, although only a minority of patients presented with shockable rhythms. The majority of IHCA survivors (~ 70%) had a good neurological outcome. Recovery of spontaneous circulation and presence of treatable acute causes of the arrest are associated with better survival.

**Graphical abstract:**

Clinical Characteristics, Causes and Predictors of Outcomes in Patients with In-Hospital Cardiac Arrest: Results from the SURVIVE-ARREST Study. Abbreviations: CPR, cardiopulmonary resuscitation; IHCA, In-hospital cardiac arrest; MCS, mechanical circulatory support; PCI, percutaneous coronary intervention; ROSC, return of spontaneous circulation; SBP, systolic blood pressure.

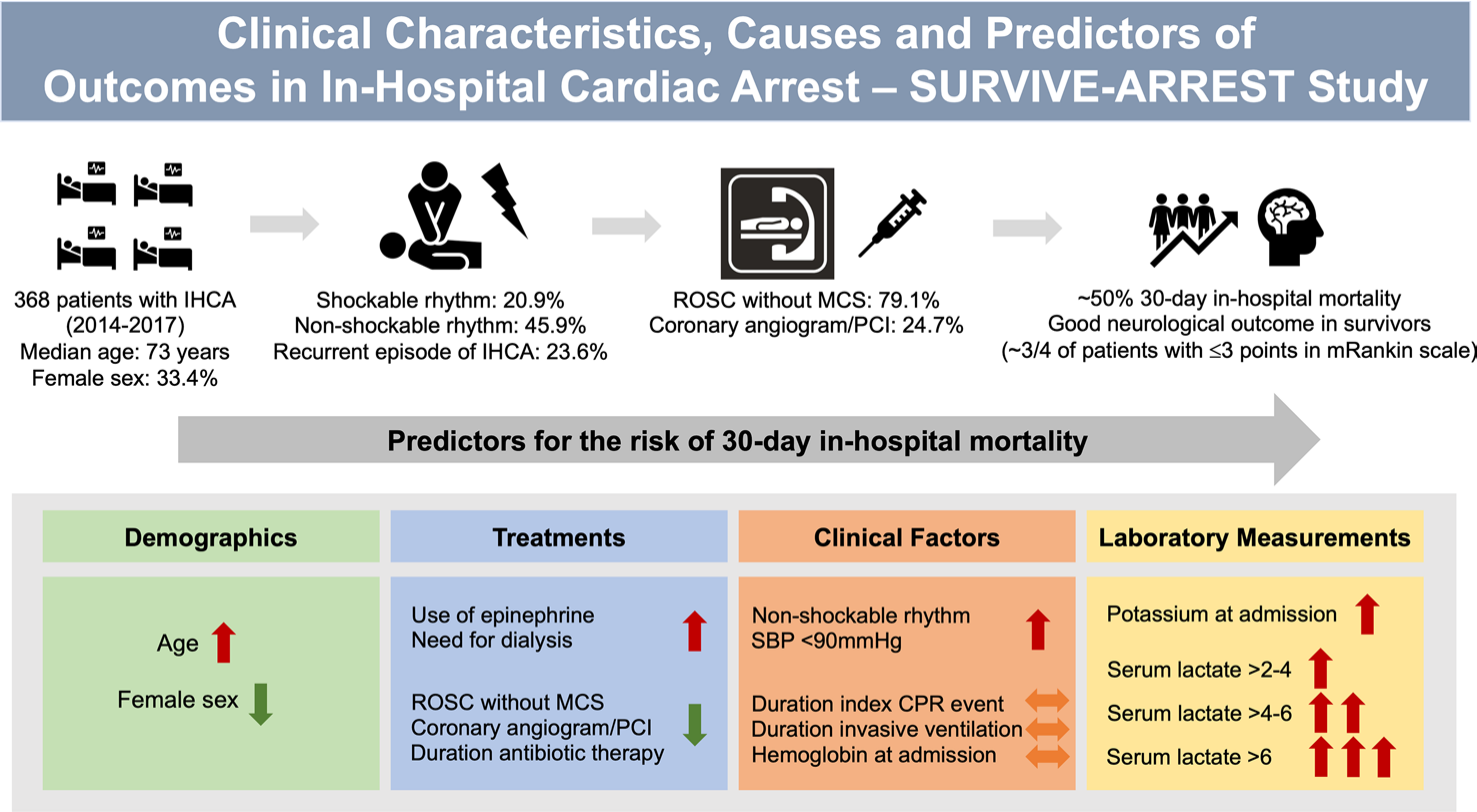

**Supplementary Information:**

The online version contains supplementary material available at 10.1007/s00392-022-02084-1.

## Introduction

In-hospital cardiac arrest (IHCA) is acutely life-threatening and remains associated with high mortality and long-term morbidity [1, 2]. Sustained return of spontaneous circulation (ROSC) is achieved in 1/3 to 2/3 of patients experiencing IHCA, and short-term survival rates after IHCA are reported to be low between 13 and 29% [3–6]. This creates a major health burden in view of the relatively high annual incidence of IHCA in Europe and the US of 1–5 per 1000 hospital admissions [6–8].

Prognostication forms the basis for decisions on treatment or withdrawal of care in patients after IHCA [9]. Identifying determinants of favourable or adverse outcomes following IHCA, therefore, seems to be of crucial importance [10]. Several clinical factors including initial rhythm, response time, CPR duration, and underlying comorbidities are related to outcomes in patients with IHCA [1, 10, 11]. Although data on outcomes after IHCA showed a considerable inter-study variability, one-year survival rates increased over time [4, 12]. Rapid initiation of cardiopulmonary resuscitation (CPR) in most patients, implementation of rapid response teams, and advanced post cardiac arrest care have been proposed as major contributors to improved survival rates in patients with IHCA [2, 13]. Over the last decade, veno-arterial extracorporeal membrane oxygenation (VA-ECMO) has evolved as a salvage therapy in patients experiencing IHCA without ROSC [14], sometimes referred to as extracorporeal cardiopulmonary resuscitation (E-CPR) [15]. Whether these recent developments and the known factors established in older cohorts still influence survival in a contemporary cohort of unselected patients presenting with IHCA is not known.

Therefore, the aim of the present study was to determine (1) patient characteristics; (2) independent predictors associated with survival after IHCA; (3) 30-day in-hospital mortality; (4) neurological outcome in patients post IHCA; and (5) discharge/referral pathways in a contemporary, unselected cohort of consecutive patients with IHCA.

## Methods

### Study population and setting

Data from the Resuscitation Registry of the University Heart and Vascular Centre Hamburg (Germany) were analysed using the Utstein-style guidelines for IHCA [16]. Consecutive patients with IHCA were enrolled and clinical data collected from the electronic health records. Death was ascertained using in-hospital records. For the current analysis, all patients with IHCA (primarily admitted to the cardiology department) between January 2014 and April 2017 were considered. Patients with prior out-of-hospital cardiac-arrest (OHCA) events and/or re-arrest after hospital admission (*n* = 56) or missing information on outcomes (*n* = 10), and patients < 18 years of age were not considered as an incident IHCA and excluded from this analysis (Supplementary Figure S1). The study was performed in accordance with the Declaration of Helsinki and the study protocol was approved by the Ethics Committee of Hamburg, Germany (PV5615). More details on the data sources are reported in the Supplementary material.

### Data collection

Data on baseline characteristics, index CPR event, post-cardiac arrest care, and outcomes were analysed. Functional neurological status after IHCA was determined through the modified Rankin Scale (mRS; range from 0 to 6 points; with 0 points representing no symptoms, 1 point no clinically significant disability, 2 points slight disability, 3 points moderate disability, 4 points moderately severe disability, 5 points severe disability, and 6 points death) at discharge [17].

### Statistical analyses

Characteristics are reported as median values with respective 25th–75th percentile for continuous data and absolute and relative frequencies for binary variables. Baseline characteristics were compared between survivors and non-survivors by Mann–Whitney *U* test (if continuous) and by chi-squared test (if categorical).

We performed competing risk analysis for the endpoint in-hospital death within 30 days considering discharge alive from hospital as competing event. Hazard Ratios (HRs) are, therefore, weighted according to the Fine & Gray estimator [18].

**Fig. 1:**
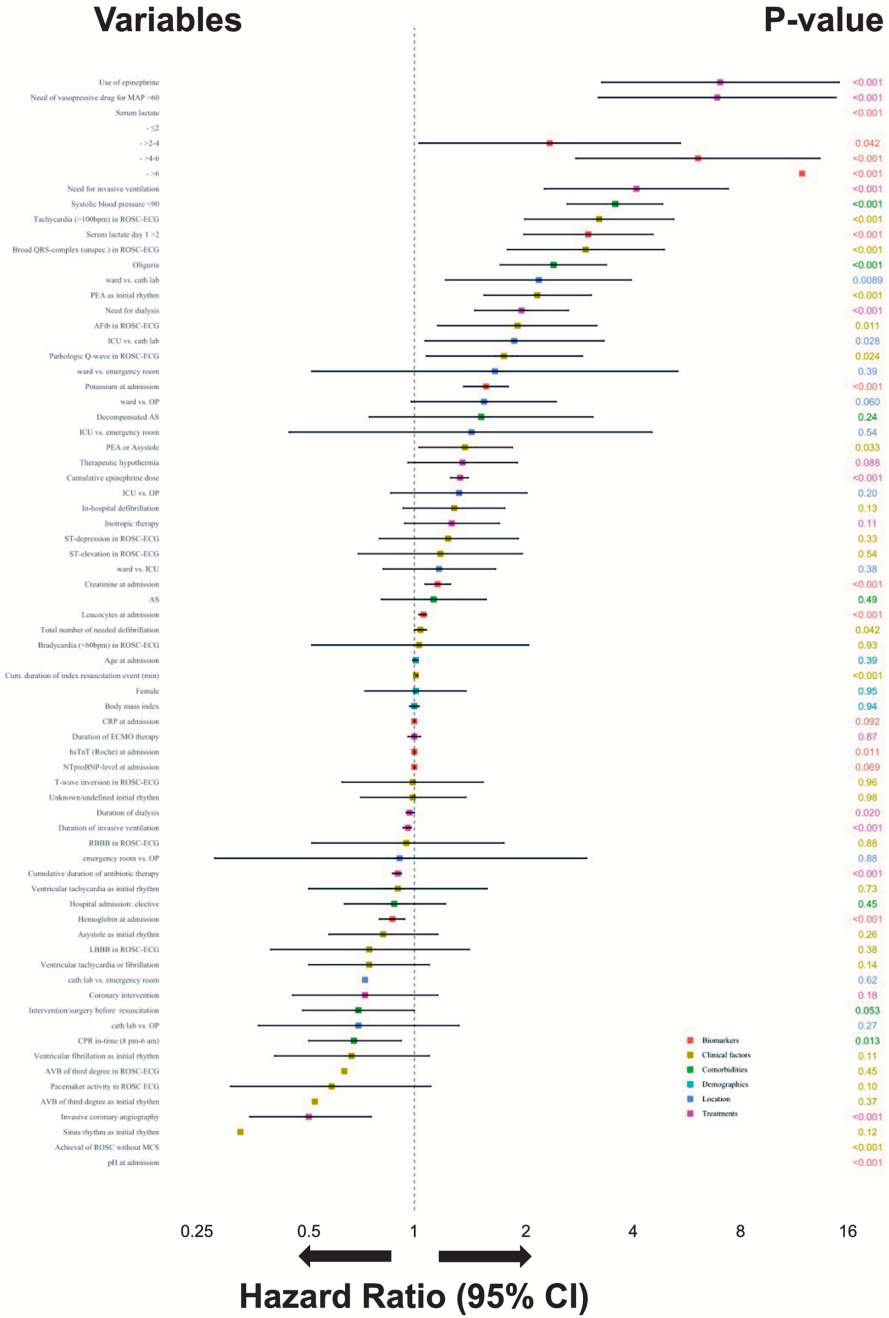
Independent predictors associated with 30-day in-hospital mortality. *Afib* atrial fibrillation, *AS* aortic stenosis, *AVB* atrioventricular block, *bpm* beats per minute, *CI* confidence interval, *CPR* C-reactive protein, *ECG* electrocardiogram, *ECMO* extracorporeal membrane oxygenation, *HR* hazard ratio, *hsTnT* high sensitivity troponin T, *ICU* intensive care unit, *LBBB* left bundle branch block, *MAP* mean arterial pressure, *MCS* mechanical circulatory support, *NT-proBNP* N-terminal-pro-brain-natriuretic peptide, *PEA* pulseless electrical activity, *pH* potential of hydrogen, *RBBB* right bundle branch block, *ROSC* return of spontaneous circulation, *SBP* systolic blood pressure, *SD* standard deviation

Univariable Cox regression models were fitted to investigate patients characteristics (demographics, clinical characteristics, comorbidities, concomitant treatments) associated with 30-day in-hospital mortality. The hazard ratios (HRs) for all univariately analysed variables and the respective 95% confidence intervals (CI) were visualized by a Forest plot (Fig. 1, Supplementary Table S2).

**Fig. 2:**
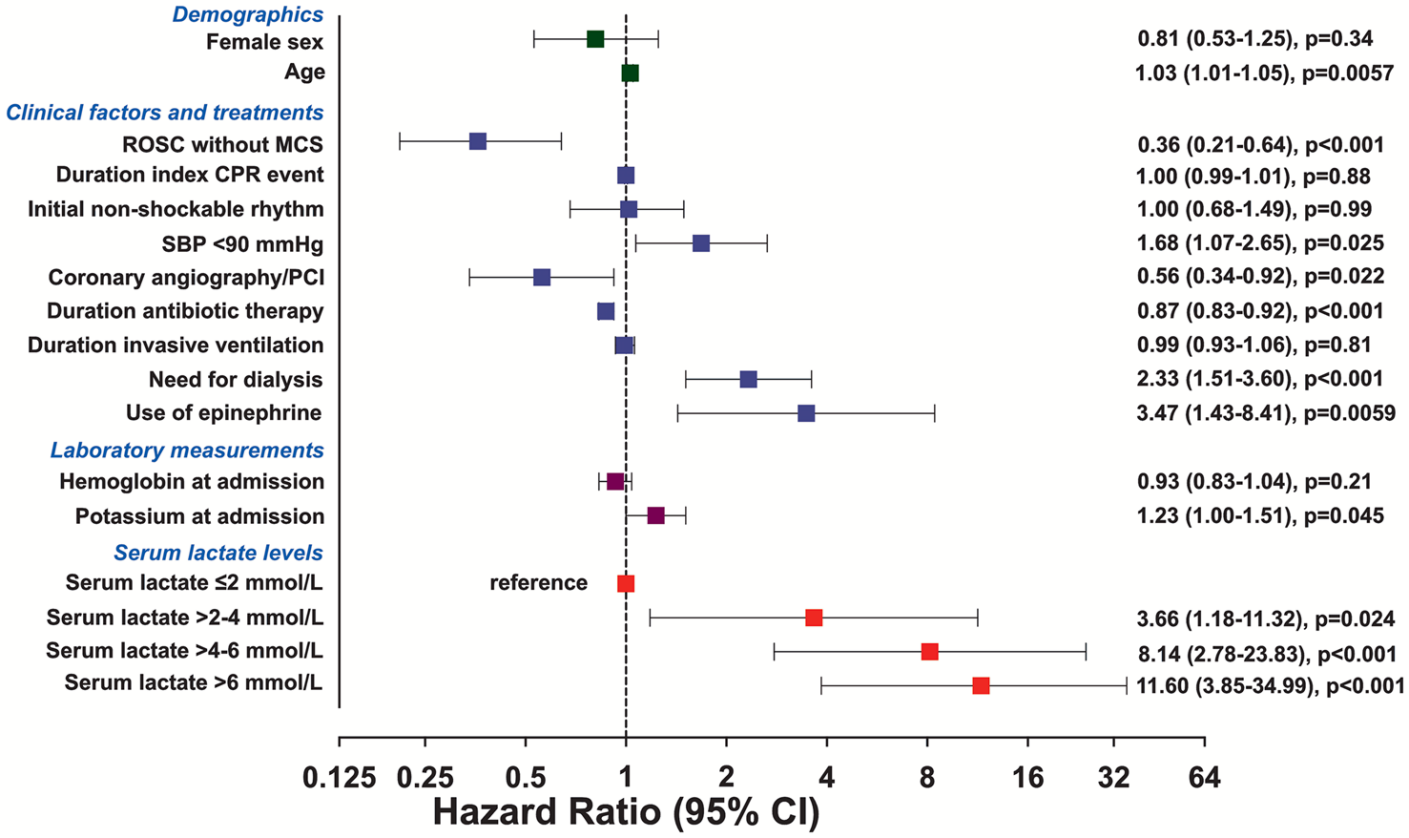
Multivariable predictors of 30-day in-hospital mortality. *CI* confidence interval, *CPR* cardiopulmonary resuscitation, *HR* hazard ratio, *MCS* mechanical circulatory support, *PCI* percutaneous coronary intervention, *ROSC* return of spontaneous circulation, *SBP* systolic blood pressure

The following variables were finally selected by clinical trade-offs for multivariable analysis: age at admission, sex, haemoglobin and potassium at admission, serum lactate levels post index CPR event in the corresponding groups: serum lactate ≤ 2 mmol/L, serum lactate > 2–4 mmol/L, serum lactate > 4–6 mmol/L, serum lactate > 6 mmol/L, cumulative duration of CPR, ROSC without MCS, non-shockable rhythm as initial rhythm in the electrocardiogram (ECG), use of epinephrine, invasive coronary angiography and/or percutaneous coronary intervention (PCI), systolic blood pressure < 90 mmHg, duration of antibiotic therapy, need for dialysis, and duration of invasive ventilation (Fig. 2, Supplementary Table S3).

Cumulative incidence curves are shown and competing risks were calculated by the Aalen–Johansen estimator. The *p*-values shown in the figures are based on Gray´s test to compare weighted averages of the subdistribution hazards across groups for the event of interest [19]. All the analyses were performed on R version 4.0.3. A *p*-value < 0.05 was considered as statistically significant.

## Results

### Patient characteristics and initial rhythm

Overall, 368 patients with IHCA were enrolled in this study from January 2014 to April 2017. The median age was 73 (interquartile range [IQR] 65–78) years and 123 (33.4%) patients were female. Cardiovascular (CV) comorbidities such as hypertension (*n* = 278, 76.4%), diabetes (*n* = 107, 29.4%), dyslipidaemia (*n* = 132, 36.3%), obstructive coronary artery disease (*n* = 253, 68.8%), and severe aortic stenosis (*n* = 96, 26.4%) were common. Modes of hospital admission were urgent admission in 109 (29.6%) patients, elective admission (e.g., for a planned intervention) in 121 (32.9%) patients, and 138 (37.5%) patients were transferred from another hospital (Graphic abstract, Table 1). The majority of IHCA was documented on intensive care unit (ICU) (*n* = 141, 41.4%) followed by ward (*n* = 94, 25.8%), operating room (*n* = 68, 18.6%), catheterization laboratory (*n* = 45, 12.3%), and emergency room (*n* = 7, 1.9%).

**Table 1:** Baseline characteristics of the study population

	All (*n* = 368)	Survivors (*n* = 196)	Non-survivors (*n* = 172)	*p* value
*Demographics*				
Age (years)	73.0 (65.0, 78.0)	73.0 (65.4, 78.6)	73.5 (65.0, 78.0)	0.83
Female sex no. (%)	123 (33.4)	67 (34.2)	56 (32.6)	0.83
BMI (kg/m^2^)	26.6 (23.6, 29.9)	26.3 (23.9, 29.4)	27.5 (23.4, 30.1)	0.63
*Comorbidities*				
Hypertension no. (%)	278 (76.4)	145 (74.4)	133 (78.7)	0.40
Smoking status no. (%)	122 (33.5)	71 (36.4)	51 (30.2)	0.25
Diabetes no. (%)	107 (29.4)	60 (30.8)	47 (27.8)	0.62
Dyslipidaemia no. (%)	132 (36.3)	70 (35.9)	62 (36.7)	0.96
CVD no. (%)	22 (6.0)	16 (8.2)	6 (3.6)	0.10
Obstructive CAD no. (%)	253 (68.8)	137 (69.9)	116 (67.4)	0.69
Severe AS	96 (26.4)	49 (25.4)	47 (27.5)	0.74
*Mode of admission to the hospital*				
Urgent admission no. (%)	109 (29.6)	57 (29.1)	52 (30.2)	0.90
Elective admission no. (%)	121 (32.9)	68 (34.7)	53 (30.8)	0.50
Transfer from other hospital no. (%)	138 (37.5)	71 (36.2)	67 (39.0)	0.67
*CPR variables*				
Cumulative duration (min)	6.0 (2.0, 17.3)	3.0 (1.0, 7.0)	15.0 (7.0, 30.6)	< 0.001
Recurrent CPR events no. (%)	87 (23.6)	28 (14.3)	59 (34.3)	< 0.001
CPR in-time (8 pm-6am) no. (%)	203 (56.5)	119 (63.0)	84 (49.4)	0.013
*CPR location*				
Ward no. (%)	94 (25.8)	43 (22.2)	51 (29.8)	0.12
ICU no. (%)	151 (41.4)	76 (39.2)	75 (43.9)	0.42
Catheterization laboratory no. (%)	45 (12.3)	31 (16.0)	14 (8.2)	0.036
Emergency room no. (%)	7 (1.9)	4 (2.1)	3 (1.8)	1.00
Operating room no. (%)	68 (18.6)	40 (20.6)	28 (16.4)	0.37
*Reason for CPR*				
STEMI no. (%)	20 (5.4)	11 (5.6)	9 (5.2)	1.00
NSTEMI no. (%)	15 (4.1)	10 (5.1)	5 (2.9)	0.42
Coronary artery dissection/perforation no. (%)	9 (2.4)	5 (2.6)	4 (2.3)	1.00
Bypass graft insufficiency/stenosis no. (%)	11 (3.0)	6 (3.1)	5 (2.9)	1.00
Aortic dissection/rupture no. (%)	6 (1.6)	2 (1.0)	4 (2.3)	0.57
Pulmonary embolism no. (%)	2 (0.5)	0.0 (0.0)	2 (1.2)	0.42
Hypoxia no. (%)	44 (12.0)	24 (12.2)	20 (11.6)	0.98
Cardiogenic shock no. (%)	88 (23.9)	34 (17.3)	54 (31.4)	0.002
Decompensated AS	14 (3.8)	6 (3.1)	8 (4.7)	0.60
Myocardial rupture/perforation no. (%)	5 (1.4)	1 (0.5)	4 (2.3)	0.29
Pericardial effusion/tamponade no. (%)	18 (4.9)	11 (5.6)	7 (4.1)	0.66
Transplant rejection no. (%)	1 (0.3)	0.0 (0.0)	1 (0.6)	0.95
Septic shock no. (%)	13 (3.5)	3 (1.5)	10 (5.8)	0.053
Volume deficiency/haemorrhagic shock no. (%)	34 (9.2)	15 (7.7)	19 (11.0)	0.35
Allergic shock no. (%)	1 (0.3)	1 (0.5)	0 (0.0)	1.0
Neurological disorder no. (%)	4 (1.1)	1 (0.5)	3 (1.7)	0.53
Metabolic disorder no. (%)	15 (4.1)	6 (3.1)	9 (5.2)	0.43
Suicide/intoxication no. (%)	1 (0.3)	1 (0.5)	0 (0.0)	1.0
*Initial rhythm*				
VF no. (%)	49 (13.3)	31 (15.8)	18 (10.5)	0.18
VT no. (%)	29 (7.9)	16 (8.2)	13 (7.6)	0.98
Asystole no. (%)	107 (29.1)	63 (32.1)	44 (25.6)	0.20
PEA no. (%)	62 (16.8)	18 (9.2)	44 (25.6)	< 0.001
AVB III° no. (%)	7 (1.9)	5 (2.6)	2 (1.2)	0.56
Sinus rhythm no. (%)	10 (2.7)	8 (4.1)	2 (1.2)	0.16
Undefined rhythm no. (%)	105 (28.5)	56 (28.6)	49 (28.5)	1.00
*Therapeutic interventions*				
Epinephrine no. (%)	295 (81.7)	133 (69.3)	162 (95.9)	< 0.001
Cumulative duration of antibiotic therapy (days)	3.0 (1.0, 10.1)	1.0 (0, 5.0)	7.0 (1.0, 12.0)	< 0.001
Coronary angiogram no. (%)	91 (24.7)	61 (31.1)	30 (17.4)	0.0036
Time to coronary angiogram (min)	55 (39.0, 111.7)	60.0 (39.9, 125.6)	54.0 (26.5, 61.0)	0.25
*First ECG after return of spontaneous circulation*				
Number with ESC after ROSC				
ST-elevation no. (%)	51 (20.2)	33 (19.0)	18 (22.8)	0.59
ST-depression no. (%)	101 (39.9)	66 (37.9)	35 (44.3)	0.41
T-wave inversion no. (%)	144 (56.9)	99 (56.9)	45 (57.0)	1.0
Pathological Q-wave no. (%)	47 (18.6)	26 (14.9)	21 (26.6)	0.042
LBBB no. (%)	45 (17.8)	34 (19.5)	11 (13.9)	0.37
RBBB no. (%)	38 (15.0)	26 (14.9)	12 (15.2)	1.0
Broad QRS-complex no. (%)	35 (13.8)	14 (8.0)	21 (26.6)	< 0.001
AFib no. (%)	45 (17.8)	25 (14.4)	20 (25.3)	0.053
Tachycardia no. (%)	40 (15.8)	15 (8.6)	25 (31.6)	< 0.001
Bradycardia no. (%)	28 (11.1)	19 (10.9)	9 (11.4)	1.0
AVB III° no. (%)	14 (5.5)	11 (6.3)	3 (3.8)	0.61
Pacemaker activity no. (%)	51 (20.2)	40 (23.0)	11 (13.9)	0.13
*MCS*				
ROSC without MCS no. (%)	291 (79.1)	188 (95.9)	103 (59.9)	< 0.001
VA-ECMO no. (%)	45 (12.2)	14 (7.1)	31 (18.0)	< 0.001
Impella® no. (%)	5 (1.4)	4 (2.0)	1 (0.6)	0.45
ECMELLA, 1 step no. (%)	9 (2.4)	4 (2.0)	5 (2.9)	0.84
ECMELLA, 2 steps no. (%)	6 (1.6)	1 (0.5)	5 (2.9)	0.16
IABP no. (%)	10 (2.7)	6 (3.1)	4 (2.3)	0.91
VAD no. (%)	7 (1.9)	1 (0.5)	6 (3.5)	0.088
*Neurological outcome*				
mRS 0–1 points	117 (62.9)	117 (62.9)	0 (0)	
mRS 2–3 points	26 (14.0)	26 (14.0)	0 (0)	
mRS 4–5 points	43 (23.1)	43 (23.1)	0 (0)	

*AFib* atrial fibrillation, *AS* aortic stenosis, *AVB* atrioventricular block, *BMI* body mass index, *CAD* coronary artery disease, *CPR* cardiopulmonary resuscitation, *CVD* cardiovascular disease, *ECMELLA* combination of veno-arterial extracorporeal life support system and the Impella® left ventricular assist device, *IABP* intra-aortic balloon pump, *ICU* intensive care unit, *LBBB* left bundle branch block, MCS mechanical circulatory support, *mRS* modified Rankin scale, *NSTEMI* non-ST-elevation myocardial infarction, *PEA* pulseless electrical activity, *ROSC* return of spontaneous circulation, *STEMI* ST-elevation myocardial infarction, *VAD* ventricular assist device, *VA-ECMO* veno-arterial extracorporeal membrane oxygenation, *VF* ventricular fibrillation, *VT* ventricular tachycardia

The most common causes of CPR were cardiogenic shock (*n* = 88, 23.9%), followed by hypoxia (*n* = 44, 12.0%), volume deficiency/haemorrhagic shock (*n* = 34, 9.2%), metabolic disorder (*n* = 15, 4.1%), and decompensated aortic stenosis (*n* = 14, 3.8%). The majority of the patients had an initial non-shockable rhythm (*n* = 169, 45.9%): 107 (29.1%) patients presented with asystole, 62 (16.8%) patients with pulseless electrical activity (PEA) as initial rhythm. Shockable rhythms were documented in 77 (20.9%) patients: 49 (13.3%) patients had ventricular fibrillation (VF), 29 (7.9%) of patients had ventricular tachycardia (VT), whereas initial detected rhythm was unknown or undefined in 105 (28.5%) patients with IHCA.

### Resuscitation and post-resuscitation information

Epinephrine was given to 295 (81.7%) patients during CPR, and initial systolic blood pressure < 90 mmHg was observed in 126 (34.4%) patients. Sustained ROSC without MCS was achieved in 79.1% (*n* = 291) and VA-ECMO was the most used MCS device (*n* = 45, 12.2%) of all implanted MCS devices in patients without ROSC. The median cumulative CPR duration was 6 (IQR 2.0–17.3) minutes. The majority of patients (*n* = 288, 79.8%) had serum lactate levels > 2 mmol/L during CPR, 282 (80.3%) patients required vasopressive medications post IHCA. Organ support in the form of invasive ventilation (*n* = 295, 80.2%) and dialysis (*n* = 128, 34.8%) were frequently required after IHCA. The median duration of ICU stay after IHCA was 6 (IQR 3.0–12.0) days (Supplementary Table S1).

### In-hospital mortality and outcome to discharge

**Fig. 3:**
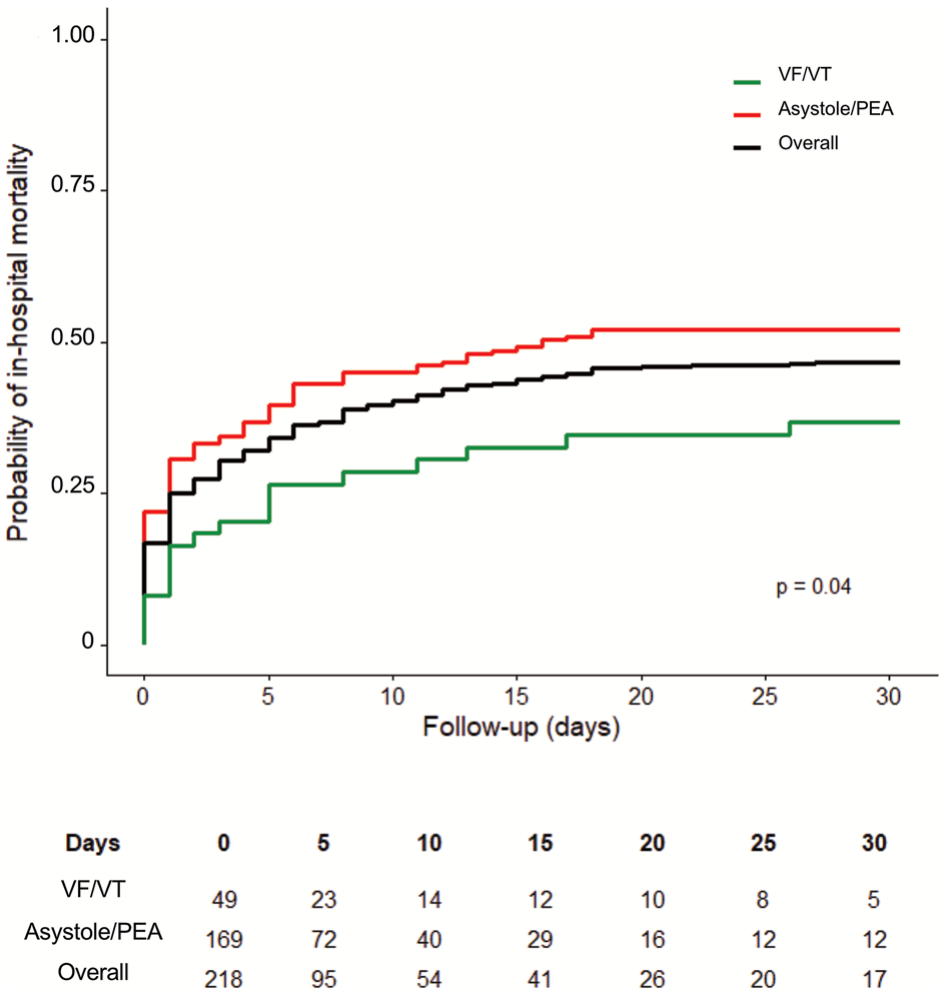
Cumulative incidence of 30-day mortality in the overall cohort and stratified by age and initial rhythm after IHCA. *PEA* pulseless electrical activity, *VF* Ventricular fibrillation, *VT* Ventricular tachycardia

**Fig. 4:**
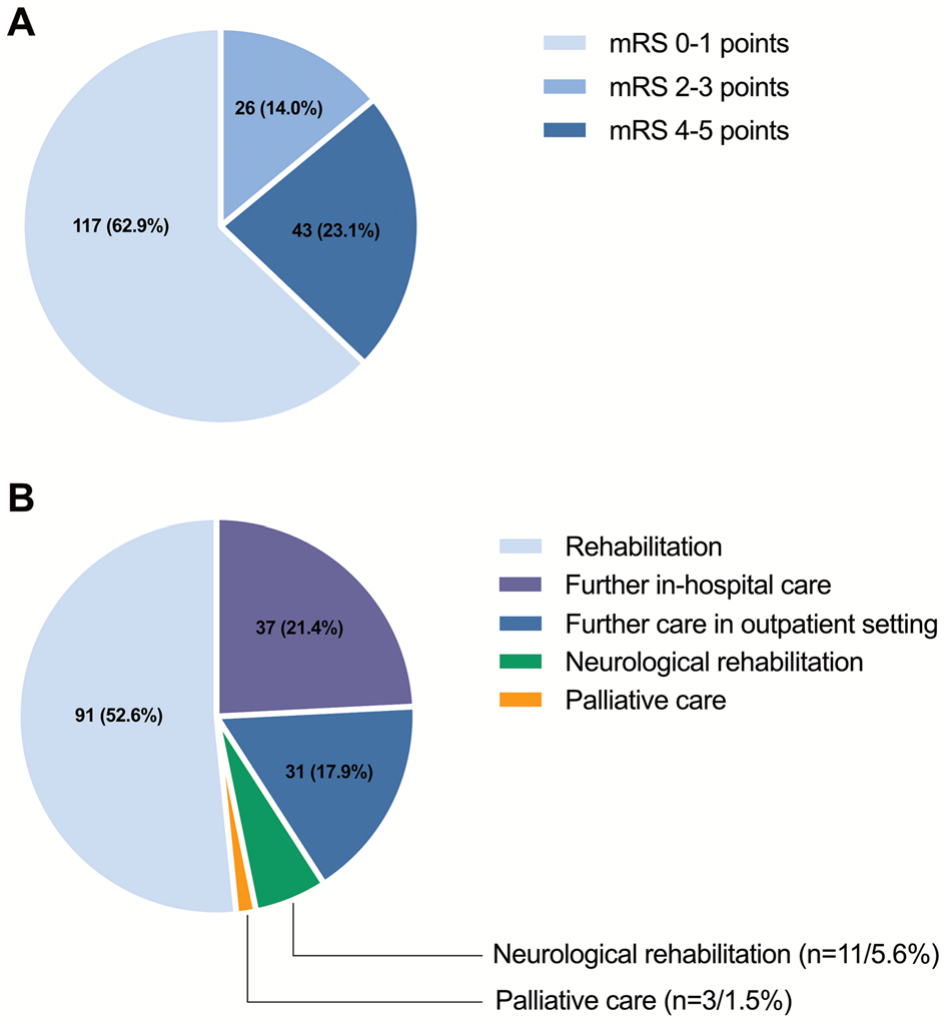
Functional neurological outcome at discharge (**A**) and discharge and referral pathways (**B**) in patients post IHCA. *mRS* modified Rankin Scale

Almost half (196/368) of the patients survived beyond 30 days after IHCA and 173 were discharged alive (Fig. 3). To assess neurological status in patients after IHCA, the mRS as a clinical outcome measure was used. Excluding the non-survivors (mRS = 6 points for death), the majority (*n* = 143, 72.9%) of survivors had a mRS ≤ 3 points, whereas only 23.1% (*n* = 43) of survivors had a mRS 4–5 points. In total, 62.9% (*n* = 117) of the survivors were discharged with none or slight disability, 14.0% (*n* = 26) with moderate disability, and 23.1% (*n* = 43) documented a severe disability 30 days post IHCA (Fig. 4A, Table 1).

About half (*n* = 96, 49.0%) of the patients 30 days post IHCA were discharged to rehabilitation. Forty-five (23.0%) patients received further in-hospital care prior to discharge home. Thirty-one (15.8%) patients post IHCA were discharged home with outpatient care. Neurological rehabilitation was required in 11 (5.6%) patients and 3 (1.5%) patients were transferred to palliative care (Fig. 4B, Supplementary Table S4).

### Patient characteristics in survivors vs. non-survivors

There were no differences in demographics (age and sex) and CV risk factors such as hypertension, diabetes, and dyslipidaemia between survivors of ICHA and those who died (Table 1). Non-survivors vs. survivors had overall longer cumulative CPR duration (median: 15 min vs. 3 min) and were more likely to have recurrent CPR events (34.3% vs. 14.3%) during hospitalization (Table 1). Survivors were less likely to have PEA (25.6% vs. 9.2%) as initial rhythm, had lower need of use of epinephrine and were more likely to have cardiogenic shock (31.4% vs. 17.3%), and to receive VA-ECMO for MCS. Other types of MCS did not differ between non-survivors and survivors. Post cardiac arrest, non-survivors had higher need of vasopressive medications, higher proportion of serum lactate levels > 2 mmol/L during resuscitation and were more likely to receive invasive ventilation, dialysis, and vasopressive therapy compared survivors (Table 1).

### Assessment of predictors of in-hospital mortality

Predictors associated with 30-day in-hospital mortality in univariate Cox regression are shown in Fig. 1. The univariate analysis identified a systolic blood pressure < 90 mmHg (HR 3.60, 95% CI 2.65–4.87; *p* < 0.001) and oliguria (HR 2.43, 95% CI 1.73–3.40; *p* < 0.001), PEA (HR 2.19, 95% CI 1.56–3.09; *p* < 0.001) or a non-shockable initial rhythm (HR 1.38, 95% CI 1.03–1.87; *p* = 0.033), tachycardia (> 100 bpm, HR 3.25, 95% CI 2.02–5.23; *p* < 0.001), a broad QRS-complex (HR 2.98, 95% CI 1.81–4.92; *p* < 0.001), atrial fibrillation (HR 1.93, 95% CI 1.16–3.20; *p* = 0.011) in the first ECG after ROSC, and a higher cumulative duration of index CPR event (HR 1.01, 95% CI 1.01–1.02; *p* < 0.001), respectively (Fig. 1, Supplementary Table S2). Treatments associated with 30-day in-hospital mortality were use and cumulative dose of epinephrine (HR 7.03, 95% CI 3.30–15.0; *p* < 0.001; HR 1.34, 95% CI 1.26–1.41; *p* < 0.001), cumulative duration of antibiotic therapy (HR 0.90, 95% CI 0.87–0.92, *p* < 0.001), and the need of vasopressive agents (HR 6.89, 95% CI 3.23–14.71; *p* < 0.001). IHCA on ward vs. catheterization laboratory was associated with a higher risk of 30-day in-hospital mortality (HR 2.21, 95% CI 1.22–3.99; *p* = 0.0089). Additionally, IHCA on ICU vs. catheterization laboratory was also associated with a higher risk of 30-day in-hospital mortality (HR 1.89, 95% CI 1.07–3.35; *p* = 0.028). All other IHCA locations were not associated with significant differences in risk of 30-day in-hospital mortality. Achievement of ROSC without MCS was associated with lower risk of 30-day in-hospital mortality (HR 0.19, 95% CI 0.14–0.26; *p* < 0.001). Furthermore, higher serum lactate > 2–4 mmol/L (HR 2.37, 95% CI 1.03–5.45; *p* = 0.042), higher serum lactate > 4–6 mmol/L (HR 6.10, 95% CI 2.80–13.27; *p* < 0.001), higher serum lactate > 6 mmol/L (HR 11.85, 95% CI 5.77–24.34; *p* < 0.001), higher serum lactate > 2 mmol/L at day 1 (HR 3.03. 95% CI 2.01–4.58, *p* < 0.001), higher potassium levels (HR 1.58, 95% CI 1.37–1.82; *p* > 0.001), lower pH levels (HR 0.02, 95% CI 0.01–0.04; *p* < 0.001), lower haemoglobin levels (HR 0.87, 95% CI 0.80–0.94; *p* < 0.001), higher leucocytes levels (HR 1.06, 95% CI 1.03–1.08; *p* < 0.001), and higher creatinine levels (HR 1.16, 95% CI 1.07–1.26; *p* < 0.001) were associated with higher risk of 30-day in-hospital mortality (Fig. 1, Supplementary Table S2).

In the multivariable Cox regression analysis, higher age, use of epinephrine, need for dialysis, duration of antibiotic therapy, higher serum lactate > 2–4 mmol/L, higher serum lactate > 4–6 mmol/L, higher serum lactate > 6 mmol/L, and higher potassium levels were associated with higher risk of 30-day in-hospital mortality (HR 1.03, 95% CI 1.01–1.05, *p* = 0.006; HR 3.47, 95% CI 1.43–8.41, *p* = 0.006; HR 2.33, 95% CI 1.51–3.60, *p* < 0.001; HR 0.87, 95% CI 0.83–0.92, *p* < 0.001; HR 3.66, 95% CI 1.18–11.32, *p* = 0.024; HR 8.14, 95% CI 2.78–23.82, *p* < 0.001; HR 11.60, 95% CI 3.85–34.99, *p* < 0.001; HR 1.23, 95% CI 1.00–1.51, *p* = 0.045, respectively) (Fig. 2, Supplementary Table S3). ROSC without MCS and use of invasive coronary angiography and/or PCI were associated with a lower risk of 30-day in-hospital mortality (HR 0.36, 95% CI 0.21–0.64, *p* < 0.001; HR 0.56, 95% CI 0.34–0.92, *p* = 0.022).

## Discussion

This analysis of a cohort of consecutive patients suffering from IHCA in a large tertiary care centre found that (1) about half of the patients survived IHCA; (2) only 1/5 (~ 20%) of patients with IHCA presented with an initial shockable rhythm; (3) ROSC without MCS, an initial shockable rhythm, antibiotic therapy, and interventions associated with an acute myocardial infarction as the cause of the arrest were associated with better survival; (4) ~ 70% of IHCA survivors had a good functional neurological outcome; and (5) the majority of patients following IHCA (> 50%) participated in (neurological) rehabilitation.

### Patient and CPR event characteristics

In our cohort, patients who experienced IHCA were characterized by a relatively high median age of 73 years, predominantly male sex, and often with known cardiovascular comorbidities such hypertension, diabetes, dyslipidaemia, coronary artery disease, and severe aortic stenosis. The age of this cohort is similar to that observed in a large UK cohort [7], and slightly lower than the median age of patients experiencing IHCA in other large data sets [6, 20]. The distribution of sexes and burden of CV comorbidities in this study is similar to the Get With The Guidelines-Resuscitation (GWTG-R) registry and National Cardiac Arrest Audit (NCAA) [7, 20].

Compared to IHCA survivors at our institution, non-survivors had an overall longer cumulative CPR duration, higher recurrent CPR event rates and were more likely to receive MCS. Consistent with results from previous studies, we found that non-survivors vs. survivors were more likely to have PEA as initial rhythm (25% vs. 8%) [21]. Interestingly, a previous study reported that individuals with cardiac arrest caused by bradyarrhythmia were more likely to be older than those with cardiac arrest caused by tachyarrhythmia [22]. Based on data from the GWTG-R registry, the cause of IHCA was most commonly cardiac followed by metabolic and respiratory [6, 23].

### In-hospital mortality, neurological outcome, and care pathways following IHCA

In our study, the 30-day in-hospital mortality of 46.7% was considerably lower than reported in previous studies [6, 7, 24, 25]. Most patients with IHCA (> 90%) were treated on monitored wards in our institution. This could have contributed to the low mortality observed compared to other studies [23].

Advanced age, CV comorbidities, duration of CPR, and non-shockable rhythms are related to a higher likelihood of in-hospital mortality in patients following IHCA [2, 26]. Advanced age has previously been associated with poor outcome after IHCA [2, 27]. Prior studies have noticed decreased post-resuscitation survival with a favourable neurological outcome in patients with a higher burden of significant CV comorbidities [9]. Importantly, hospitalized patients with a high burden of significant CV comorbidities reflect a more vulnerable patient population associated with a higher incidence of IHCA [28]. Less aggressive treatment and a worse risk profile might contribute to these findings. We also found that ~ 20% of patients with IHCA had an initial shockable rhythm, lower than in recent other studies reporting a lower survival rate [12, 29]. Taking other results into account, the proportion of patients with IHCA and initial shockable rhythms was reported to decreased from 31.6% in 2008 to 23.6% in 2018 [12]. Although IHCA-related mortality, especially in the setting of PEA/asystole, decreased during the last two decades, we found higher 30-day in-hospital mortality in patients with IHCA and initial PEA/asystole compared to patients with IHCA and initial VF/VT. This is of particular interest since the proportion of hospitalized patients with PEA/asystole is expected to increase and highlights future challenges for health care professionals treating these critically ill patients [12, 20]. In our study, we found that a remarkably high proportion (~ 25%) of patients with IHCA had comorbid severe aortic stenosis. This might be explained by the fact that the investigated patients in this study were primarily referred to the cardiology department, e.g., for valve treatment. Interestingly, comorbid severe aortic valve stenosis was not associated with a higher risk of 30-day in-hospital mortality and only a small proportion of patients with IHCA suffered from decompensated aortic stenosis as reason for IHCA (~ 4%). While others identified aortic valve stenosis as independent predictor for mortality, the prognostic value of aortic stenosis in IHCA is still under investigation [30].

Physical, cognitive and social health care problems impacting negatively quality of life are frequently observed in patients post IHCA [31]. Notably, most treatment recommendations for patients with IHCA are based on studies of patients after OHCA [32]. Neurocognitive impairment is the most observed neurological sequelae for all patients post cardiac arrest in the early stages and roughly ~ 50% in the long term [33]. In our study, the majority of IHCA survivors had a good functional neurological outcome (~ 70% of patients with mRS ≤ 3 points), whereas > 50% of the patients post IHCA had a need for (neurological) rehabilitation. These findings highlight the need for and the importance of patient-centred rehabilitation in this vulnerable patient population [34].

### Clinical factors related to in-hospital mortality in patients with IHCA

We identified several clinical factors independently associated with 30-day in-hospital mortality in patients who experienced IHCA. The present study showed that IHCA survivors had a significantly higher likelihood of having a shockable rhythm as first recorded rhythm compared to non-survivors. This observation is in line with those of previous studies also demonstrating that patients with IHCA and a non-shockable rhythm have poorer survival to hospital discharge than patients with IHCA and a shockable rhythm [11]. In our analysis, we found that the risk of 30-day in-hospital mortality increased with elevated serum lactate levels (initial measurements and subsequent measurements at day 1 post IHCA) in patients suffering from IHCA. This is in line with previous studies reporting improved survival rates in patients with better lactate clearance after IHCA [35]. Additionally, lower initial systolic blood pressure < 90 mmHg was identified as an independent predictor of poor survival after IHCA.

Cardiac arrest caused by acute myocardial infarction was associated with an improved survival derived by the potential for more effective treatment like invasive coronary angiography and/or PCI as a surrogate marker of acute myocardial infarction as we have documented in our study [6]. In addition, our study revealed that cardiac arrest location ward or ICU vs. catheterization laboratory was associated with a higher risk of 30-day in-hospital mortality (HR 2.21, 95% CI 1.22–3.99; *p* = 0.0089 and HR 1.89, 95% CI 1.07–3.35; *p* = 0.028, respectively) suggesting successful PCI among patients resuscitated from acute coronary syndrome or shockable rhythms during PCI. We also confirmed the overall impact of prompt initiation of CPR and early achievement of sustained ROSC on outcomes in patients with cardiac arrest. Compared to previous studies, MCS as bridge to decision was used in ~ 10% of patients post IHCA at our institution, which might be due to the overall high availability and experience in utilization of MCS [13]. Despite the increasing use of MCS, mortality of patients requiring MCS is still high, and there are only a few randomized data demonstrating no clear benefit of this therapeutic option. Appropriate patient selection using risk prediction tools/models (e.g., the SAVE and ENCOURAGE score) are keys to maximizing the benefit and minimizing the risk of severe complications [36, 37].

In contrast to other recent studies, we found a lower risk of 30-day in-hospital mortality during night hours in comparison with events that occurred during the dayshift [38]. The importance of sex to outcomes has attracted some attention, with previous studies showing better survival in female vs. male patients post IHCA [26]. In the present study, sex did not appear as a significant predictor of 30-day in-hospital mortality in patients following IHCA.

### Limitations

Our study should be interpreted in the context of several limitations. Due to the retrospective and single-centre character of our study, there is a risk of confounding and selection bias. This study includes an undefined initial rhythm in 28.5% of patients following IHCA and residual confounding on post CPR treatments. Moreover, we did not provide information concerning low-flow vs. no-flow time during resuscitation. Another limitation of this study represents its locoregional pathology as a single-centre retrospective study. Reporting from a highly specialized centre for CV care and high-volume cardiac arrest centre, our results may be not generally transferable to other clinical settings. Specifically, the quality of care and outcomes may be different than in other hospitals. By only taking IHCA cases of our institution (tertiary hospital) into account the study and its heterogeneity is reduced. The effects of a rapid CPR response, overall high monitoring rate in our institution (> 90%), and the high availability of MCS may bias individualized outcomes (e.g., overall high survival rates of ~ 50% within 30 days after IHCA in our study). In multivariable analysis, we could face the problem of overfitting, but all included variables were assessed as important. Finally, there are uncertainties regarding the time intervals that were measured.

## Conclusion

In this contemporary cohort of patients with IHCA, 30-day in-hospital mortality was ~ 50% in a tertiary care hospital. An initial shockable rhythm was found in ~ 20% of patients. Key predictors of a lower 30-day in-hospital mortality risk were achievement of ROSC without MCS, antibiotic therapy, and the need for interventions associated with an acute myocardial infarction as the cause of the arrest. The majority of IHCA survivors (~ 70%) had a good functional neurological outcome. Further studies are needed to develop new strategies to increase awareness and to improve monitoring and care of hospitalized patients.

## Supplementary Information

Below is the link to the electronic supplementary material.

Supplementary file1 (DOCX 77 KB)

## Data Availability

The data underlying this article will be shared on reasonable request to the corresponding author.

## Abbreviations

CPR: Cardiopulmonary resuscitation
CRP: C-reactive protein
CI: Confidence interval
CV: Cardiovascular
ECG: Electrocardiogram
E-CPR: Extracorporeal cardiopulmonary resuscitation
Hs-cTnT: High-sensitivity troponin T
ICU: Intensive care unit
IHCA: In-hospital cardiac arrest
IQR: Interquartile range
HR: Hazard ratio
MCS: Mechanical circulatory support
NT-proBNP: N-terminal pro-B-type natriuretic peptide
OHCA: Out-of-hospital cardiac arrest
PEA: Pulseless electrical activity
PCI: Percutaneous coronary intervention
ROSC: Return of spontaneous circulation
SBP: Systolic blood pressure
US: United States
VA-ECMO: Veno-arterial extracorporeal membrane oxygenation
