# Supplementary material for: Clinical characteristics, causes and predictors of outcomes in patients with in-hospital cardiac arrest: results from the SURVIVE-ARREST study
Source: Clin Res Cardiol. 2022 Aug 17;112(2):258–69. doi: 10.1007/s00392-022-02084-1 (PMC9898362; doi:10.1007/s00392-022-02084-1)
Supplement: Supplementary file 1 — Supplementary file1 (DOCX 77 KB) [file 392_2022_2084_MOESM1_ESM.docx]

**Supplementary Material**

Supplementary Material to: Hannen et al., Clinical Characteristics, Causes and Predictors of Outcomes in Patients with In-Hospital Cardiac Arrest: Results from the SURVIVE-ARREST Study

**Table of Contents**   **Page number**

**Supplementary Material**

**Supplementary Material.** 2

Data collection.

**Supplementary Figure**

**Supplementary Figure S1.** 3

Flow-chart of the study population

**Supplementary Tables**

**Supplementary Table S1.** 4

Post-cardiac arrest care data of all IHCA cases

between January 2014 and April 2017

**Supplementary Table S2.** 6

Independent predictors associated with in-hospital mortality

**Supplementary Table S3.** 8

Multivariable predictors of in-hospital mortality

**Supplementary Table S4.** 9

Referral/discharge pathways in IHCA survivors

between January 2014 and April 2017

**Supplementary Material.**

*Data Collection*

Baseline characteristics included age, sex, comorbidities, mode of/reason for admission to the hospital and presence of any intervention or surgery before index CPR (***Table 1***). Data on the index CPR events comprised duration of CPR, information on recurrent CPR events, reason for CPR, initial rhythm, locations of index CPR event, diagnostic/therapeutic interventions, use of mechanical circulatory support (MCS), and characteristics of the first electrocardiogram recorded after ROSC (***Table 1***). Post-cardiac arrest data were extracted from electronic intensive care unit (ICU) records: duration of ICU stay, vital parameters at admission to ICU, and therapeutic interventions/medications during ICU stay (***Supplementary Table S1***). Laboratory measurements including blood gas analysis, blood count, renal parameters, liver enzymes, C-reactive protein (CRP), N-terminal pro-B-type natriuretic peptide (NT-proBNP), and high-sensitivity troponin T (hs-cTnT) were recorded at ICU admission and at first day of ICU stay (initial measurements and subsequent measurements at day 1 post IHCA) *(****Supplementary Table S1****).*

**Supplementary Figure S1.** Flow-chart of the study population

Abbreviations: IHCA, In-hospital cardiac arrest; OHCA, Out-of-hospital cardiac arrest.

**Supplementary Table S1.** Post-cardiac arrest care data of all IHCA cases between January 2014 and April 2017

| **Supplementary Table S1** | | | | |
| --- | --- | --- | --- | --- |
|  | **All**  **(n=368)** | **Survivors**  **(n=196)** | **Non-survivors**  **(n=172)** | **p-value** |
| ***Clinical factors*** | | | | |
| Duration of ICU stay (days) | 6.0 (3.0, 12.0) | 7.0 (4.0, 17.0) | 5.0 (2.0, 11.0) | <0.001 |
| SBP <90 mmHg no. (%) | 126 (34.4) | 36 (18.4) | 90 (52.9) | <0.001 |
| Need of vasopressive drugs for MAP >60 mmHg no. (%) | 282 (80.3) | 134 (68.4) | 148 (95.5) | <0.001 |
| Serum-lactate >2 mmol/L no. (%) | 288 (79.8) | 129 (66.5) | 159 (95.2) | <0.001 |
| ***Therapeutic interventions*** | | | | |
| Dialysis no. (%) | 128 (34.8) | 44 (22.4) | 84 (48.8) | <0.001 |
| Invasive ventilation no. (%) | 295 (80.2) | 135 (68.9) | 160 (93.0) | <0.001 |
| Inotropic therapy no. (%) | 140 (38.0) | 64 (32.7) | 76 (44.2) | 0.030 |
| Norepinephrine no. (%) | 301 (82.0) | 151 (77.0) | 150 (87.7) | 0.012 |
| Cumulative duration of antibiotic therapy (days) | 3.0 (1.0, 10.1) | 8.0 (1.0, 16.0) | 1.0 (0.0, 5.0) | <0.001 |
| ***Laboratory measures*** | | | | |
| Serum-lactate (mmol/L) | 5.0 (2.4, 8.8) | 3.0 (1.5, 5.5) | 7.9 (4.9, 11.9) | <0.001 |
| Serum-lactate > 2 (mmol/L) no. (%) | 288 (79.8) | 129 (66.5) | 159 (95.2) | <0.001 |
| Serum-lactate ≤ 2 (mmol/L) no. (%) | 73 (20.2) | 65 (33.5) | 8 (4.8) | <0.001 |
| Serum-lactate > 2-4 (mmol/L) no. (%) | 76 (21.1) | 58 (29.9) | 18 (10.8) | <0.001 |
| Serum-lactate > 4-6 (mmol/L) no. (%) | 62 (17.2) | 31 (16.0) | 31 (18.6) | 0.61 |
| Serum-lactate > 6 (mmol/L) no. (%) | 150 (41.6) | 40 (20.6) | 110 (65.9) | <0.001 |
| pH (mmol/L) | 7.3 (7.2, 7.4) | 7.3 (7.2, 7.4) | 7.2 (7.1, 7.3) | <0.001 |
| Haemoglobin (g/dL) | 9.8 (8.6, 11.1) | 9.9 (8.9, 11.5) | 9.4 (8.2, 10.8) | 0.0025 |
| Leucocytes (Mrd/L) | 13.0 (9.0, 18.5) | 11.3 (7.9, 15.0) | 16.1 (11.1, 21.6) | <0.001 |
| Potassium (mmol/L) | 4.4 (3.9, 5.0) | 4.2 (3.8, 4.7) | 4.6 (4.1, 5.4) | <0.001 |
| Creatinine (mg/dL) | 1.6 (1.3, 2.4) | 1.4 (1.1, 2.1) | 2.1(1.5, 2.9) | <0.001 |
| CRP (mg/L) | 32.5 (8.0, 100.1) | 17.0 (7.0, 81.1) | 53.5 (11.0, 119.7) | 0.0095 |
| NT-proBNP (ng/L) | 3181.5  (518.3, 18065.1) | 2094.0 (413.9, 16847.8) | 8985.5 (1638.8, 46520.1) | 0.16 |
| Hs-TnT (ng/mL) | 434.0  (140.3, 1555.2) | 353.5  (107.7, 1238.2) | 638.0  (224.3, 2022.7) | 0.0087 |
| ***Laboratory measures day 1*** | | | | |
| Serum-lactate (mmol/L) day 1 | 1.4 (1.0, 2.2) | 1.2 (0.9, 1.7) | 1.9 (1.2, 3.4) | <0.001 |
| Serum-lactate day 1 ≥2 (mmol/L) no. (%) | 81 (28.4) | 38 (19.6) | 43 (47.3) | <0.001 |
| pH (mmol/L) day 1 | 7.4 (7.4, 7.5) | 7.4 (7.4, 7.5) | 7.4 (7.3, 7.5) | 0.066 |
| Haemoglobin (g/dL) day 1 | 9.7 (8.8, 10.8) | 9.7 (8.8, 10.8) | 9.6 (8.6, 10.8) | 0.44 |
| Leucocytes (Mrd/L) day 1 | 10.2 (7.7, 14.6) | 9.6 (7.6, 12.9) | 12.5 (7.7, 18.0) | 0.004 |
| Potassium (mmol/L) day 1 | 4.2 (3.9, 4.5) | 4.1 (3.9, 4.4) | 4.3 (4.0, 4.6) | 0.035 |
| Creatinine (mg/dL) day 1 | 1.5 (1.1, 2.2) | 1.3 (1.1, 1.9) | 1.9 (1.4, 2.6) | <0.001 |
| CRP (mg/L) day 1 | 70.0 (28.0, 142.3) | 64.0 (22.2, 130.5) | 81.0 (45.0, 145.2) | 0.017 |
| NT-proBNP (ng/L) day 1 | 1108.5 (421.7, 4656.6) | 4163.5 (1205.0, 7122.0) | 506.0 (0.1, 1012.0) | 0.12 |
| Hs-TnT (ng/mL) day 1 | 903.0 (369.2, 2407.2) | 783.5 (270.6, 1892.0) | 1751.0 (573.8, 6177.7) | 0.002 |

**Abbreviations:** CRP: C-reactive protein; hs-TnT: high sensitivity troponin T; ICU: intensive care unit; MAP: mean arterial pressure; NT-proBNP: N-terminal-pro-brain-natriuretic peptide; pH: potential of hydrogen; SBP: systolic blood pressure.

**Supplementary Table S2.** Independent predictors associated with in-hospital mortality

| Supplementary Table S2 |  | |
| --- | --- | --- |
|  | **HR (95% CI)** | **p-value** |
| ***Demographics*** | | |
| Age | 1.01 (0.99, 1.02) | 0.39 |
| Female sex | 1.01 (0.73, 1.39) | 0.95 |
| Body mass index | 1.00 (0.97, 1.03) | 0.94 |
| ***Comorbidities*** | | |
| SBP <90 mmHg | 3.60 (2.65, 4.87) | <0.001 |
| Oliguria (urine output less than 400 mL/day) | 2.43 (1.73, 3.40) | <0.001 |
| CPR in-time (8pm-6am) | 0.68 (0.51, 0.92) | 0.013 |
| Intervention/surgery before resuscitation | 0.70 (0.49, 1.00) | 0.053 |
| Severe AS | 1.13 (0.81, 1.58) | 0.49 |
| Decompensated severe AS | 1.53 (0.75, 3.12) | 0.24 |
| ***Clinical factors*** | | |
| Cumulative duration of index resuscitation event (min.) | 1.01 (1.01, 1.02) | <0.001 |
| In-hospital defibrillation | 1.29 (0.93, 1.78) | 0.13 |
| Total number of needed defibrillations to ROSC | 1.04 (1.00, 1.08) | 0.042 |
| Achievement of ROSC without MCS | 0.19 (0.14, 0.26) | <0.001 |
| Sinus rhythm as initial rhythm | 0.33 (0.08, 1.34) | 0.12 |
| Asystole as initial rhythm | 0.82 (0.58, 1.16) | 0.26 |
| Ventricular fibrillation as initial rhythm | 0.67 (0.41, 1.10) | 0.11 |
| Ventricular tachycardia as initial rhythm | 0.90 (0.51, 1.59) | 0.73 |
| PEA as initial rhythm | 2.19 (1.56, 3.09) | <0.001 |
| AVB III° in ROSC-ECG | 0.64 (0.20, 2.02) | 0.45 |
| Non-shockable rhythm | 1.38 (1.03, 1.87) | 0.033 |
| Shockable rhythm | 0.75 (0.51, 1.10) | 0.14 |
| Unknown/undefined initial rhythm | 0.99 (0.71, 1.39) | 0.98 |
| AFib in ROSC-ECG | 1.93 (1.16, 3.20) | 0.011 |
| Tachycardia (>100bpm) in ROSC-ECG | 3.25 (2.02, 5.23) | <0.001 |
| Bradycardia (<60bpm) in ROSC-ECG | 1.03 (0.52, 2.07) | 0.93 |
| Broad QRS-complex (unspecified) in ROSC-ECG | 2.98 (1.81, 4.92) | <0.001 |
| Pathologic Q-wave in ROSC-ECG | 1.77 (1.08, 2.92) | 0.024 |
| ST-elevation in ROSC-ECG | 1.18 (0.70, 1.99) | 0.54 |
| ST-depression in ROSC-ECG | 1.24 (0.80, 1.94) | 0.33 |
| T-wave inversion of ROSC-ECG | 0.99 (0.63, 1.55) | 0.96 |
| RBBB in ROSC-ECG | 0.95 (0.52, 1.77) | 0.88 |
| LBBB in ROSC-ECG | 0.75 (0.40, 1.42) | 0.38 |
| Pacemaker activity in ROSC-ECG | 0.59 (0.31, 1.11) | 0.10 |
| AVB III° as initial rhythm | 0.53 (0.20, 2.02) | 0.45 |
| ***Treatments*** | | |
| Need for use of epinephrine | 7.03 (3.30, 15.00) | <0.001 |
| Cumulative epinephrine dose | 1.34 (1.26, 1.41) | <0.001 |
| Need for inotropic therapy | 1.27 (0.94, 1.72) | 0.11 |
| Need for vasopressive drug for MAP >60 mmHg | 6.89 (3.23, 14.71) | <0.001 |
| Need for invasive ventilation | 4.12 (2.29, 7.40) | <0.001 |
| Duration of invasive ventilation | 0.96 (0.93, 0.98) | <0.001 |
| Need for dialysis | 1.98 (1.47, 2.67) | <0.001 |
| Duration of dialysis | 0.97 (0.95, 1.00) | 0.020 |
| Duration of ECMO therapy | 1.00 (0.96, 1.04) | 0.87 |
| Cumulative duration of antibiotic therapy | 0.90 (0.87, 0.92) | <0.001 |
| Percutaneous coronary intervention | 0.73 (0.46, 1.16) | 0.18 |
| Invasive coronary angiography | 0.51 (0.35, 0.76) | <0.001 |
| ***Laboratory measurements*** | | |
| Serum-lactate ≤2 mmol/L | 1 (reference) |  |
| Serum-lactate >2-4 mmol/L | 2.37 (1.03, 5.45) | 0.042 |
| Serum-lactate >4-6 mmol/L | 6.10 (2.80, 13.27) | <0.001 |
| Serum-lactate >6 mmol/L | 11.85 (5.77, 24.34) | <0.001 |
| Serum-lactate >2 mmol/L at day 1 | 3.03 (2.01, 4.58) | <0.001 |
| Potassium at admission | 1.58 (1.37, 1.82) | <0.001 |
| Creatinine at admission | 1.16 (1.07, 1.26) | <0.001 |
| pH at admission | 0.02 (0.01, 0.04) | <0.001 |
| Leucocytes at admission | 1.06 (1.03, 1.08) | <0.001 |
| CRP at admission | 1.00 (1.00, 1.00) | 0.092 |
| NT-proBNP at admission | 1.00 (1.00, 1.00) | 0.069 |
| hsTnT at admission | 1.00 (1.00, 1.00) | 0.011 |
| Haemoglobin at admission | 0.87 (0.80, 0.94) | <0.001 |
| ***Location CPR*** |  |  |
| Ward vs. catheterization laboratory | 1.89 (1.07, 3.35) | 0.028 |
| Ward vs. emergency room | 1.67 (0.52, 5.36) | 0.39 |
| Ward vs. operating room | 1.56 (0.98, 2.47) | 0.06 |
| Ward vs. ICU | 1.17 (0.82, 1.68) | 0.38 |
| ICU vs. catheterization laboratory | 2.21 (1.22, 3.99) | 0.009 |
| ICU vs. emergency room | 1.44 (0.45, 4.55) | 0.54 |
| ICU vs. operating room | 1.33 (0.86, 2.05) | 0.20 |
| Emergency room vs. operating room | 0.91 (0.28, 3.00) | 0.88 |
| Catheterization laboratory vs. emergency room | 0.73 (0.21, 2.54) | 0.62 |
| Catheterization laboratory vs. operating room | 0.70 (0.37, 1.33) | 0.27 |

**Abbreviations:** AFib: atrial fibrillation; AS: aortic stenosis; AVB: atrioventricular block; CI: confidence interval; CPR: cardiopulmonary resuscitation; CRP: C-reactive protein; ECG: electrocardiogram; ECMO: extracorporeal membrane oxygenation; HR: hazard ratio; hsTnT: high sensitivity troponin T; ICU: intensive care unit; LBBB: left bundle branch block; MAP: mean arterial pressure; MCS: mechanical circulatory support; NT-proBNP: N-terminal-pro-brain-natriuretic peptide; PEA: pulseless electrical activity; pH: potential of hydrogen; RBBB: right bundle branch block; ROSC: return of spontaneous circulation; SBP: systolic blood pressure; SD: standard deviation.

**Supplementary Table S3.** Multivariable predictors of 30-day in-hospital mortality

| Supplementary Table S3 | | |
| --- | --- | --- |
|  | **HR (95% CI)** | **p-value** |
| ***Demographics*** | | |
| Female sex | 0.81 (0.53, 1.25) | 0.34 |
| Age | 1.03 (1.01, 1.05) | 0.006 |
| ***Clinical factors*** | | |
| Achievement of ROSC without MCS | 0.36 (0.21, 0.64) | <0.001 |
| Duration of index resuscitation event | 1.00 (0.99, 1.01) | 0.88 |
| Non-shockable rhythm as initial rhythm | 1.00 (0.68, 1.49) | 0.99 |
| Systolic blood pressure <90 mmHg | 1.68 (1.07, 2.65) | 0.0025 |
| ***Treatments*** | | |
| Invasive coronary angiography/PCI | 0.56 (0.34, 0.92) | 0.022 |
| Cumulative duration of antibiotic therapy | 0.87 (0.83, 0.92) | <0.001 |
| Duration of invasive ventilation | 0.99 (0.93, 1.06) | 0.81 |
| Need for dialysis | 2.33 (1.51, 3.60) | <0.001 |
| Use of epinephrine | 3.47 (1.43, 8.41) | 0.006 |
| ***Laboratory measurements*** |  |  |
| Haemoglobin at admission | 0.91 (0.82, 1.01) | 0.083 |
| Potassium at admission | 1.32 (1.08, 1.60) | 0.006 |
| Serum-lactate ≤2 mmol/L | 1 (reference) |  |
| Serum-lactate >2-4 mmol/L | 3.66 (1.18, 11.32) | 0.024 |
| Serum-lactate >4-6 mmol/L | 8.14 (2.78, 23.83) | <0.001 |
| Serum-lactate >6 mmol/L | 11.60 (3.85, 34.99) | <0.001 |

**Abbreviations:** CI, confidence interval; HR, hazard ratio; MCS, mechanical circulatory support; PCI, percutaneous coronary intervention; ROSC, return of spontaneous circulation.

**Supplementary Table S4.** Referral/discharge pathways in IHCA survivors between January 2014 and April 2017

| **Supplementary Table S4** |  |
| --- | --- |
| **Referral/discharge pathways** | **Survivors (n=196)** |
| Rehabilitation no. (%) | 96 (49.0) |
| Further in-hospital care no. (%) | 45 (23.0) |
| Further outpatient care no. (%) | 31 (15.8) |
| Neurological rehabilitation no. (%) | 11 (5.6) |
| Palliative care no. (%) | 3 (1.5) |
